# Collaboration between health sciences librarians and faculty as reflected by articles published in the *Journal of the Medical Library Association*

**DOI:** 10.5195/jmla.2018.559

**Published:** 2018-10-01

**Authors:** Katherine G. Akers, Molly Higgins, Jennifer A. DeVito, Sally Stieglitz, Robert Tolliver, Clara Y. Tran

**Affiliations:** Editor-in-Chief, *Journal of the Medical Library Association*, and Biomedical Research and Data Specialist, Shiffman Medical Library, Wayne State University, Detroit, MI; Reference and Digital Services Librarian, Library of Congress, Washington, DC; Director of Access and User Services, Frank Melville Jr. Memorial Library, Stony Brook University, Stony Brook, NY; Digital Learning and Instruction Librarian, Swirbul Library, Adelphi University, Garden City, NY; Head, Science and Engineering, Frank Melville Jr. Memorial Library, Stony Brook University, Stony Brook, NY; Science Librarian, Frank Melville Jr. Memorial Library, Stony Brook University, Stony Brook, NY

## Abstract

A recent study by Higgins and colleagues reports that the *Journal of the Medical Library Association (JMLA)* had the highest percentage of articles with both librarian and faculty coauthors out of 13 peer-reviewed journals in science, technology, engineering, and medicine librarianship and education between 2005 and 2014. A deeper and updated analysis of *JMLA* research articles and case studies published between 2008 and 2017 revealed that 29% of articles had both librarian and faculty coauthors. The main topics of librarian-faculty collaboration, as described in these articles, were related to patient and consumer health information and clinical information-seeking and decision-making by health care providers. Most faculty coauthors came from the disciplines of biomedical or health informatics and biostatistics and library and information science. The publication of these articles in the *JMLA* provides evidence of health sciences librarians’ and information specialists’ ability to collaborate with faculty members to advance the knowledgebase and practice of librarianship and the health sciences.

## INTRODUCTION

Last year, Higgins and colleagues found that out of 13 peer-reviewed journals in science, technology, engineering, and medicine (STEM) librarianship and education, the *Journal of the Medical Library Association (JMLA)* had the highest percentage (12%) of articles with both librarian and faculty coauthors between 2005 and 2014 [1]. They also found that more than half of the examined articles appeared in health sciences journals (including *Health Information and Libraries Journal* and *Medicine Reference Services Quarterly*) as opposed to science and engineering journals, suggesting that “medical librarians as a professional group have been accepted by medical faculty and clinicians within research, education, and clinical teams” [1].

As the editor of the *JMLA,* I was thrilled to see the journal at the top of this list, which provided evidence of health sciences librarians’ and information specialists’ ability to collaborate with faculty members to advance the knowledgebase and practice of librarianship and the health sciences. At the same time, however, some questions came to mind: What are the topics of these collaborative articles? What are the most common disciplines of collaborating faculty members? To answer these questions, I asked Higgins and her coauthors to share their data [2], they said “yes”!, and I set about performing additional analyses over a more recent time span (between 2008 and 2017).

## LIBRARIAN-FACULTY COLLABORATIVE ARTICLES IN THE *JOURNAL OF THE MEDICAL LIBRARY ASSOCIATION*

The line between being a librarian versus being a faculty member can be fuzzy. For the purpose of this analysis, I defined “librarian” as practicing librarians, library directors, full-time library staff, informationists, information specialists, curators, archivists, National Library of Medicine staff and fellows, or Medical Library Association staff, regardless of whether their positions were tenure-track. I defined “faculty” as nonpractioner professors in library and information science (LIS), professors in any other non-LIS discipline, or physicians. Data visualization was performed using Tableau software [3].

Between 2008 and 2017, I found that 29% (n=109) of research articles and case studies published in the *JMLA* (n=374) had both librarian and faculty coauthors, whereas 59% (n=222) and 13% (n=50) of such articles had only librarian authors or no librarian authors, respectively. That is, early a third of *JMLA* articles describing research projects or library-driven initiatives were borne out of collaboration between librarians and faculty members or physicians.

The proportion of librarian-faculty collaborative articles published in the *JMLA* remained fairly stable across this 10-year period despite some year-to-year variation (range, 15%–42%), and the number of article coauthors—regardless of their identity—also held steady over time, with an average of 3 authors per article. The much higher proportion of collaborative articles found in this analysis compared with the previous analysis by Higgins and colleagues [1] (29% versus 12%) might be due to several factors, including LIS professors being considered faculty and published conference symposia being excluded in the present analysis.

## TOPICS OF LIBRARIAN-FACULTY COLLABORATIVE ARTICLES

**Figure 1:**
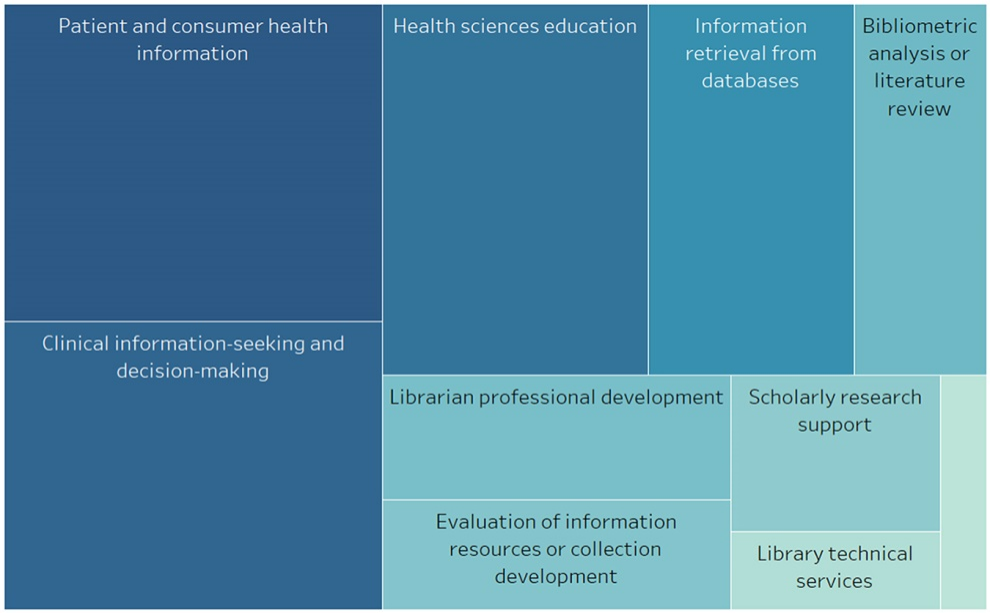
Treemap of the topics of *Journal of the Medical Library Association (JMLA)* articles with both librarian and faculty coauthors

To identify the topic areas of librarian-faculty collaborations as described by *JMLA* articles, I categorized each collaborative article by its primary focus. I found that the main topics of collaboration were related to patient and consumer health information (n=22) and clinical information-seeking and decision-making by health care providers (n=20) (Figure 1). Other topics, in descending order of frequency, pertained to health sciences education (n=18), information retrieval from databases (n=14), bibliometric analysis or literature reviews (n=9), librarian professional development (n=8), evaluation of health information resources or library collection development (n=7), scholarly research support services (n=6), library technical services (n=3), and other (n=2). Thus, in contrast to Higgins and colleagues’ finding that instruction and information literacy were the most common topics of articles published across all STEM journals [1], the collaborative articles published in the *JMLA* pertain largely to connecting health care consumers and health care providers to medical information to improve the delivery of health care.

## DISCIPLINES OF FACULTY COLLABORATORS

**Figure 2:**
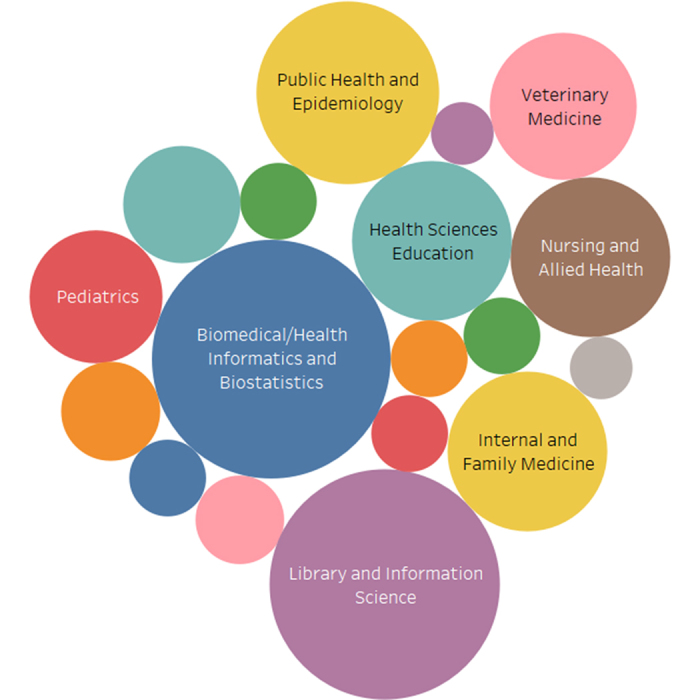
Bubble chart of the disciplines of faculty coauthors of *JMLA* articles

To identify the disciplines of the 167 faculty coauthors of the 109 collaborative *JMLA* articles, I classified individual faculty members based on their primary departmental affiliations as provided in the articles or, if not available, professional information discerned through Google searches. Perhaps not surprisingly, I found that most faculty coauthors came from the disciplines of biomedical or health informatics and biostatistics (n=29) and LIS (n=27) (Figure 2). Other common disciplines of faculty coauthors were public health and epidemiology (n=17), health sciences education (n=13), internal and family medicine (n=13), nursing and allied health (n=13), veterinary medicine (n=11), and pediatrics (n=9). Less common but still participating disciplines were pharmacy and pharmacology (n=7), neurology and neurosurgery (n=4), cell and molecular biology (n=3), emergency medicine (n=3), health care delivery and policy (n=3), ophthalmology and optometry (n=3), psychiatry (n=3), oncology (n=2), surgery (n=2), and other (n=5).

## CONCLUSIONS

The results of the present analysis and that of Higgins and colleagues [1] highlight the important work that health sciences librarians are doing in partnership with faculty members and physicians. These collaborative efforts have the beneficial outcomes of helping patients and health care consumers access and become more enlightened users of health-related information, helping health care providers obtain and use medical information to aid their clinical decision-making, enhancing medical and health sciences students’ ability to find and evaluate health-related information, and improving the retrieval of information from health-related databases.

Importantly, these analyses do not touch upon the undoubtedly large body of literature reflecting librarian-faculty collaborations that appear in medical, information science, and other types of journals [4] that also advance health sciences education and practice. For instance, 30% of systematic reviews published in high-impact medical journals have librarian coauthors or acknowledge librarian involvement, with those systematic reviews involving librarians being associated with higher-quality literature searches [5] and presumably higher-quality medical evidence as a consequence.

To increase the capability and readiness of health sciences librarians and information specialists to work alongside faculty and physicians as members of interdisciplinary research teams, the profession must continue to provide opportunities for research training and mentorship (e.g., the MLA Research Training Institute for Health Sciences Librarians) and to formalize institutional support for research engagement in terms of providing funding, protecting research time, and fostering a “research culture” in libraries [6]. Also, librarians can increase the likelihood of finding collaborating faculty members by leaving the physical confines of their libraries, serving on campus-wide committees, attending nonlibrary conferences and workshops, publishing in nonlibrary journals, and even pursuing another advanced subject degree [7].

Finally, congratulations to those health sciences librarians and information specialists who have successfully forged partnerships with faculty or physicians and published the outcomes. Keep up the good work!
